# Supplementary material for: The Systems Biology Research Tool: evolvable open-source software
Source: BMC Syst Biol. 2008 Jun 29;2:55. doi: 10.1186/1752-0509-2-55 (PMC2446383; doi:10.1186/1752-0509-2-55)
Supplement: Additional file 1 — SBRT Archive. An archive of the current version of the Systems Biology Research Tool. [file 1752-0509-2-55-S1.zip › sbrt-1.4.0/doc/developers_guide/api/index-files/index-6.html]

F-Index


|  |  |  |
| --- | --- | --- |
| **Overview** \| Package \| Class \| Use \| **Tree** \| **Deprecated** \| **Index** \| **Help** | |  |
| **PREV LETTER**   **NEXT LETTER** | **FRAMES**    **NO FRAMES**     **All Classes** |  |


A B C D E F G H I J K L M N O P Q R S T U V W X Y Z 

---


## **F**

**FastMsFluxRangeComputer** - Class in sbrt.kernel.fba.comp: This class is used to compute Mahadevan-Schilling flux intervals in a (sometimes) faster way than `MsFluxIntervalComputer` can. **FastMsFluxRangeComputer(FbaOptComputer, Map<String, Set<LinearComb<String>>>, LinearSystemSolution<String>, double)** - Constructor for class sbrt.kernel.fba.comp.FastMsFluxRangeComputer: Constructs a new flux interval computer. **FatalException** - Exception in sbrt: This class is used to represent an unrecoverable, or fatal, exception. **FatalException(String)** - Constructor for exception sbrt.FatalException: Constructs a new object. **FbaManager** - Class in sbrt.shell.mng.fba: This abstract class provides a foundation for Flux Balance Analysis managers. **FbaManager()** - Constructor for class sbrt.shell.mng.fba.FbaManager: **FbaOptComputer** - Class in sbrt.kernel.fba.comp: This class is the workhorse of all Flux Balance Analysis optimizations. **FbaOptComputer(IrrevRxnFluxome, ProgramSolver<Object, Object>)** - Constructor for class sbrt.kernel.fba.comp.FbaOptComputer: Constructs a new FBA optimization computer from the provided fluxome and program solver. **FbaOptComputer(IrrevRxnFluxome, ProgramSolver<Object, Object>, double)** - Constructor for class sbrt.kernel.fba.comp.FbaOptComputer: Constructs a new FBA optimization computer from the provided fluxome, program solver, and constraint tolerance. **FbaOptComputer.Results** - Class in sbrt.kernel.fba.comp: This class is used to store the results of an FBA optimization problem. **FbaOptException** - Exception in sbrt.kernel.fba.comp: This class is used to indicate that an optimal solution could not be found or does not exist. **FbaOptException(MathExpr<?>, OptimizationSense, ProgramSolverStatus)** - Constructor for exception sbrt.kernel.fba.comp.FbaOptException: Constructs a new FBA optimization exception. **FbaOptException(MathExpr<?>, OptimizationSense, ProgramSolverStatus, String)** - Constructor for exception sbrt.kernel.fba.comp.FbaOptException: Constructs a new FBA optimization exception. **FbaOptFileLineFormat** - Interface in sbrt.shell.text.fba: This interface is used to represent formats of FBA optimization file lines. **FbaOptFileLineFormatV1** - Class in sbrt.shell.text.fba: This class is a concrete implementation of `FbaOptFileLineFormat`. **FbaOptFileLineFormatV1(Fluxome)** - Constructor for class sbrt.shell.text.fba.FbaOptFileLineFormatV1: Constructs a new FBA optimization file line format. **FbaOptHeaderFormatter** - Interface in sbrt.shell.text.fba: This interface is used to represent formatters for headers used in writing files of FBA optimization data. **FbaOptHeaderFormatterV1** - Class in sbrt.shell.text.fba: This class is a concrete implemenation of `FbaOptHeaderFormatter`. **FbaOptHeaderFormatterV1(Fluxome)** - Constructor for class sbrt.shell.text.fba.FbaOptHeaderFormatterV1: Constructs a new FBA optimization header formatter. **FbaOptHeaderParser** - Interface in sbrt.shell.text.fba: This interface is used to represent parsers for headers used in writing files of FBA optimization data. **FbaOptHeaderParserV1** - Class in sbrt.shell.text.fba: This class is a concrete implemenation of `FbaOptHeaderParser`. **FbaOptHeaderParserV1(Fluxome)** - Constructor for class sbrt.shell.text.fba.FbaOptHeaderParserV1: Constructs a new FBA optimization header parser. **FbaOptimizationProcess** - Class in sbrt.kernel.fba.proc: This class is used to perform basic Flux Balance Analysis optimization. **FbaOptimizationProcess(FbaOptComputer)** - Constructor for class sbrt.kernel.fba.proc.FbaOptimizationProcess: Constructs a new FBA optimization experiment. **FbaOptManager** - Class in sbrt.shell.mng.fba: This class is used to manage basic FBA optimization experiments. **FbaOptManager()** - Constructor for class sbrt.shell.mng.fba.FbaOptManager: **FbaOptOutputFile** - Interface in sbrt.shell.io.fba: This interface is used to represent FBA optimization output files. **FbaProcess**<R> - Class in sbrt.kernel.fba.proc: This abstract class is used to represent Flux Balance Analysis experiments. **FbaProcess()** - Constructor for class sbrt.kernel.fba.proc.FbaProcess: **FbaSolver** - Class in sbrt.kernel.fba.comp: This class is used to solve the system of linear equations represented by *Sv = 0*. **FbaSolver()** - Constructor for class sbrt.kernel.fba.comp.FbaSolver: **FbaSolverManager** - Class in sbrt.shell.mng.fba: This class is used to manage FBA solver processes. **FbaSolverManager()** - Constructor for class sbrt.shell.mng.fba.FbaSolverManager: **FbaSolverProcess** - Class in sbrt.kernel.fba.proc: This class is used to solve Sv = 0. **FbaSolverProcess(Fluxome)** - Constructor for class sbrt.kernel.fba.proc.FbaSolverProcess: Constructs a new FBA solver process. **FILE** - Static variable in class sbrt.shell.mng.utilities.VectorComparisonManager: The string used to indicate the name of the vector file. **FILE\_1** - Static variable in class sbrt.shell.mng.utilities.IntervalComparisonManager: The string used to indicate the name of the first file. **FILE\_2** - Static variable in class sbrt.shell.mng.utilities.IntervalComparisonManager: The string used to indicate the name of the second file. **FILE\_FORMAT** - Static variable in class sbrt.shell.mng.AbstractProcessManager: The keyword used to denote the name of a file format. **FileFormat** - Class in sbrt.shell.io: This class is used to represent file formats. **FileFormatFormat** - Class in sbrt.shell.text: This class is used to represent formats for types of file formats. **FileFormatFormat()** - Constructor for class sbrt.shell.text.FileFormatFormat: **FileLineIterator**<E> - Class in sbrt.shell.io: This class is used to iterate through the lines of a file. **FileLineIterator()** - Constructor for class sbrt.shell.io.FileLineIterator: **finalize()** - Method in class sbrt.ext.mma.algebra.MathematicaSolver: Calls `close()`. **finalize()** - Method in class sbrt.shell.io.SerializedFileIterator: Disposes of system resources associated with this file iterator when called by the garbage collector. **finalize()** - Method in class sbrt.shell.io.SerializedInputFile: Disposes of system resources associated with this file when called by the garbage collector. **finalize()** - Method in class sbrt.shell.io.SerializedOutputFile: Disposes of system resources associated with this file when called by the garbage collector. **finalize()** - Method in class sbrt.shell.io.TextFileLineIterator: Disposes of system resources associated with this file iterator when called by the garbage collector. **finalize()** - Method in class sbrt.shell.io.TextInputFile: Disposes of system resources associated with this file when called by the garbage collector. **finalize()** - Method in class sbrt.shell.io.TextOutputFile: Disposes of system resources associated with this file when called by the garbage collector. **fixedVariables()** - Method in class sbrt.kernel.math.algebra.HashLinearSystemSolution: Returns the number of fixed variables in this solution. **fixedVariables()** - Method in interface sbrt.kernel.math.algebra.LinearSystemSolution: Returns the number of fixed variables in this solution. **FLUX\_BOUNDS\_FILE\_NAME** - Static variable in class sbrt.shell.mng.fba.PalssonSbmlReaderManager: Used to denote the name of the file to which the flux bounds should be written. **FLUX\_CAP\_FILE** - Static variable in class sbrt.shell.mng.fba.MsFluxIntervalManager: The keyword used to denote the flux cap input file. **FLUX\_INTERVAL\_FILE** - Static variable in class sbrt.shell.mng.fba.FluxPlasticityManager: The keyword used to denote the name of the file containing the sets of flux intervals. **FLUX\_INTERVAL\_FILE** - Static variable in class sbrt.shell.mng.fba.InitialPointManager: The keyword used to denote the name of the file containing the set of flux intervals. **FLUX\_INTERVAL\_FILE** - Static variable in class sbrt.shell.mng.fba.IntervalVariationManager: The keyword used to denote a flux interval input file. **FLUX\_VECTOR\_FILE** - Static variable in class sbrt.shell.mng.fba.FbaManager: The keyword used to denote a flux vector input file. **FLUX\_VECTOR\_FILE\_FORMAT** - Static variable in class sbrt.shell.mng.fba.FluxActivityManager: The keyword used to denote the format of the flux vector input file. **FLUX\_VECTOR\_FILE\_NAME** - Static variable in class sbrt.shell.mng.fba.FbaManager: The keyword used to denote the name of a flux vector output file. **FLUX\_VECTOR\_FILE\_NAME** - Static variable in class sbrt.shell.mng.fba.PalssonSbmlReaderManager: Used to denote the name of the file to which the flux vector should be written. **FluxActivityManager** - Class in sbrt.shell.mng.fba: This class is used to manage flux activity experiments. **FluxActivityManager()** - Constructor for class sbrt.shell.mng.fba.FluxActivityManager: **FluxActivityProcess** - Class in sbrt.kernel.fba.proc: This class is used to perform flux activity experiments. **FluxActivityProcess(Fluxome)** - Constructor for class sbrt.kernel.fba.proc.FluxActivityProcess: Constructs a new flux activity experiment. **FluxActivityProcess.Results** - Class in sbrt.kernel.fba.proc: This class is used to represent the results of a flux activity experiment. **FluxCapComputer** - Class in sbrt.kernel.fba.comp: This class is used to compute *flux caps* for use in Mahadevan-Schilling flux interval computations. **FluxCapComputer(Fluxome, DoubleMatrix<?, String>)** - Constructor for class sbrt.kernel.fba.comp.FluxCapComputer: Constructs a new flux cap computer from the cycles contained in the provided fluxome. **FluxCapComputer(Fluxome, Collection<LinearComb<String>>)** - Constructor for class sbrt.kernel.fba.comp.FluxCapComputer: Constructs a new flux cap computer from the cycles contained in the provided fluxome. **FluxCapException** - Exception in sbrt.kernel.fba.comp: This class is used to indicate that a flux cap is invalid. **FluxCapException(MathExpr, String, Throwable)** - Constructor for exception sbrt.kernel.fba.comp.FluxCapException: Constructs a new flux cap exception. **FluxCapException(MathExpr, String)** - Constructor for exception sbrt.kernel.fba.comp.FluxCapException: Constructs a new flux cap exception. **FluxCapFormat** - Interface in sbrt.shell.text.fba: This interface is used to represent the formats of lines of flux cap-containing files. **FluxCapFormatV1** - Class in sbrt.shell.text.fba: This class is a concrete implementation of `FluxCapFormat`. **FluxCapFormatV1(Fluxome)** - Constructor for class sbrt.shell.text.fba.FluxCapFormatV1: Constructs a new flux cap file line format using the provided fluxome. **FluxCapInputFile** - Class in sbrt.shell.io.fba: This class is used to read files containing flux caps. **FluxCapInputFile(String, FluxCapFormat)** - Constructor for class sbrt.shell.io.fba.FluxCapInputFile: Constructs a new flux cap input file. **FluxCapManager** - Class in sbrt.shell.mng.fba: This class is used to manage flux cap experiments. **FluxCapManager()** - Constructor for class sbrt.shell.mng.fba.FluxCapManager: **FluxCapProcess** - Class in sbrt.kernel.fba.proc: This class is used to perform a flux cap identification. **FluxCapProcess(FluxCapComputer)** - Constructor for class sbrt.kernel.fba.proc.FluxCapProcess: Constructs a new flux cap process. **FluxConstraints** - Class in sbrt.kernel.fba: This class is used to group a set of flux constraints used in Flux Balance Analysis. **FluxConstraints(Map<String, Interval>, Map<? extends MathExpr<String>, Interval>)** - Constructor for class sbrt.kernel.fba.FluxConstraints: Constructs a set of FBA constraints from the provided arguments. **FluxConstraints(Map<String, Interval>)** - Constructor for class sbrt.kernel.fba.FluxConstraints: Constructs a set of FBA constraints from the provided argument. **FluxCvComputer** - Interface in sbrt.kernel.fba.comp: This interface is used to represent a flux constraint variation computer. **FluxIntervalAnalyzer** - Class in sbrt.kernel.fba.analysis: This class is used to analyze flux intervals. **FluxIntervalAnalyzer()** - Constructor for class sbrt.kernel.fba.analysis.FluxIntervalAnalyzer: Constructs a new flux interval analyzer. **FluxIntervalComputer** - Interface in sbrt.kernel.fba.comp: This interface is used to represent flux interval computers. **FluxIntervalInputFile** - Class in sbrt.shell.io.fba: This class is used to read flux interval input files. **FluxIntervalInputFile(String, FileFormat, Set<LinearComb<String>>, MapFormat<LinearComb<String>, Interval>)** - Constructor for class sbrt.shell.io.fba.FluxIntervalInputFile: Constructs a new flux-interval input file. **FluxIntervalProcess** - Class in sbrt.kernel.fba.proc: This class is used to perform flux interval experiments. **FluxIntervalProcess(FluxIntervalComputer)** - Constructor for class sbrt.kernel.fba.proc.FluxIntervalProcess: Constructs a new flux range experiment. **Fluxome** - Interface in sbrt.kernel.fba: This interface is used to represent fluxomes for use in Flux Balance Analysis. **FluxomeReducer**<T extends Fluxome> - Interface in sbrt.kernel.fba.comp: This interface is used to represent algorithms that reduce the size of fluxomes. **FluxomeReducerProcess**<T extends Fluxome> - Class in sbrt.kernel.fba.proc: This class is used to execute fluxome reduction processes. **FluxomeReducerProcess(T, FluxomeReducer<T>)** - Constructor for class sbrt.kernel.fba.proc.FluxomeReducerProcess: Constructs a new fluxome reduction process. **FluxomeSolFileLineFormatV1** - Class in sbrt.shell.text.fba: This class is a concrete implemenation of `FluxomeSolutionFileLineFormat`. **FluxomeSolFileLineFormatV1(Fluxome)** - Constructor for class sbrt.shell.text.fba.FluxomeSolFileLineFormatV1: Constructs a new fluxome solution file line format. **FluxomeSolutionFileLineFormat** - Interface in sbrt.shell.text.fba: This interface is used to represent formats for solutions to the linear system of equations formed by a fluxome. **FluxPlasticityManager** - Class in sbrt.shell.mng.fba: This class is used to manage flux plasticity experiments. **FluxPlasticityManager()** - Constructor for class sbrt.shell.mng.fba.FluxPlasticityManager: **FluxPlasticityProcess** - Class in sbrt.kernel.fba.proc: This class is used to perform flux plasticity experiments. **FluxPlasticityProcess(Fluxome)** - Constructor for class sbrt.kernel.fba.proc.FluxPlasticityProcess: Constructs a new flux plasticity experiment. **FluxPlasticityProcess.Results** - Class in sbrt.kernel.fba.proc: This class is used to represent the results of a flux plasticity experiment. **FluxRangeException** - Exception in sbrt.kernel.fba.comp: This class is used to indicate that the range of flux interval has exceeded a defined cutoff value. **FluxRangeException(double, Object, String)** - Constructor for exception sbrt.kernel.fba.comp.FluxRangeException: Constructs a new flux range exception. **FluxRangeException(double, Object)** - Constructor for exception sbrt.kernel.fba.comp.FluxRangeException: Constructs a new flux range exception. **FluxVectorFormat** - Interface in sbrt.shell.text.fba: This interface is used to represent formats for flux vectors. **FluxVectorFormatV1** - Class in sbrt.shell.text.fba: This class is a concrete implemenation of `FluxVectorFormat`. **FluxVectorFormatV1(Fluxome)** - Constructor for class sbrt.shell.text.fba.FluxVectorFormatV1: Constructs a new flux vector format. **format(Set<? extends E>)** - Method in class sbrt.shell.text.AbstractLinkedHashSetFormat: Returns a formatted string representation of the provided collection. **format(List<? extends E>)** - Method in class sbrt.shell.text.AbstractListFormat: Returns a formatted string representation of the provided collection. **format(Boolean)** - Method in interface sbrt.shell.text.BooleanFormat: Returns a formatted string representation of the provided boolean value. **format(boolean)** - Method in interface sbrt.shell.text.BooleanFormat: Returns a formatted string representation of the provided boolean value. **format(Or<And<E>>)** - Method in class sbrt.shell.text.BoolRelFormatV1: Returns a formatted string representation of the provided boolean relationship. **format(String)** - Method in class sbrt.shell.text.chem.CatalystFormatV1: Returns the provided catalyst name after ensuring it conforms to this format. **format(String)** - Method in class sbrt.shell.text.chem.CatalystFormatV2: Returns the provided catalyst name after ensuring it conforms to this format. **format(String)** - Method in class sbrt.shell.text.chem.ChemSpeciesFormatV1: Returns the provided chemical species name after ensuring it conforms to this format. **format(IrrevRxn)** - Method in class sbrt.shell.text.chem.IrrevRxnFormatV1: Returns a formatted string representation of the provided irreversible chemical reaction. **format(IrrevRxn)** - Method in class sbrt.shell.text.chem.IrrevRxnFormatV2: Returns a formatted string representation of the provided irreversible chemical reaction. **format(ReversibleRxn)** - Method in class sbrt.shell.text.chem.RevRxnFormatV1: Returns a formatted string representation of the provided reversible chemical reaction. **format(ReversibleRxn)** - Method in class sbrt.shell.text.chem.RevRxnFormatV2: Returns a formatted string representation of the provided reversible chemical reaction. **format(String)** - Method in class sbrt.shell.text.chem.RxnNameFormatV1: Returns the provided reaction name after ensuring it conforms to this format. **format(Collection<? extends E>)** - Method in interface sbrt.shell.text.CollectionFormat: Returns a formatted string representation of the provided collection. **format(Double)** - Method in interface sbrt.shell.text.DoubleFormat: Returns a formatted string representation of the provided double precision number. **format(double)** - Method in interface sbrt.shell.text.DoubleFormat: Returns a formatted string representation of the provided double precision number. **format(Double)** - Method in class sbrt.shell.text.DoubleFormatV1: Returns a formatted string representation of the provided double precision number. **format(double)** - Method in class sbrt.shell.text.DoubleFormatV1: Parses the provided string and returns its corresponding double precision number. **format(Double)** - Method in class sbrt.shell.text.DoubleFormatV2: Returns a formatted string representation of the provided double precision number. **format(double)** - Method in class sbrt.shell.text.DoubleFormatV2: Parses the provided string and returns its corresponding double precision number. **format(List<String>)** - Method in interface sbrt.shell.text.fba.CatalystListFormat: Returns a formatted string representation of the provided list of catalyst names. **format(List<String>)** - Method in class sbrt.shell.text.fba.CatalystListFormatV1: Returns a formatted string representation of the provided list of catalyst names. **format(Set<String>)** - Method in interface sbrt.shell.text.fba.CatalystSetFormat: Returns a formatted string representation of the provided set of enzyme names. **format(Set<String>)** - Method in class sbrt.shell.text.fba.CatalystSetFormatV1: Returns a formatted string representation of the provided set of catalyst names. **format(String)** - Method in class sbrt.shell.text.fba.CatalystVerifier: Returns the provided catalyst name after ensuring it exists in the corresponding fluxome of this catalyst name verifier. **format(String)** - Method in class sbrt.shell.text.fba.ChemSpeciesVerifier: Returns the provided chemical species after ensuring it exists in the corresponding fluxome of this chemical species verifier. **format(String)** - Method in class sbrt.shell.text.fba.FbaOptHeaderFormatterV1: Returns a formatted string representation of the provided header. **format(ChemRxn)** - Method in class sbrt.shell.text.fba.IrrevRxnFormatV1: Returns a formatted string representation of the provided irreversible chemical reaction. **format(IrrevRxn)** - Method in class sbrt.shell.text.fba.IrrevRxnFormatV1: Returns a formatted string representation of the provided irreversible chemical reaction. **format(SourceRxn)** - Method in class sbrt.shell.text.fba.IrrevRxnFormatV1: Returns a formatted string representation of the provided source. **format(SinkRxn)** - Method in class sbrt.shell.text.fba.IrrevRxnFormatV1: Returns a formatted string representation of the provided sink. **format(LinearComb<String>)** - Method in class sbrt.shell.text.fba.RxnNameExprFormatV1: Returns a formatted string representation of the provided linear combination of reaction names. **format(List<String>)** - Method in interface sbrt.shell.text.fba.RxnNameListFormat: Returns a formatted string representation of the provided list of reaction names. **format(List<String>)** - Method in class sbrt.shell.text.fba.RxnNameListFormatV1: Returns a formatted string representation of the provided list of reaction names. **format(Object)** - Method in class sbrt.shell.text.fba.RxnNameOrExprFormatV1: Returns the string representation of the provided object. **format(Set<String>)** - Method in interface sbrt.shell.text.fba.RxnNameSetFormat: Returns a formatted string representation of the provided set of reaction names. **format(Set<String>)** - Method in class sbrt.shell.text.fba.RxnNameSetFormatV1: Returns a formatted string representation of the provided set of reaction names. **format(String)** - Method in class sbrt.shell.text.fba.RxnNameVerifier: Returns the provided reaction name after ensuring it exists in the corresponding fluxome of this reaction name checker. **format(FileFormat)** - Method in class sbrt.shell.text.FileFormatFormat: Returns a formatted string representation of the provided file format. **Format** - Interface in sbrt.shell.text: This interface is used to represent formats. **format(S)** - Method in interface sbrt.shell.text.Formatter: Returns a formatted string representation of the provided object. **format(Integer)** - Method in interface sbrt.shell.text.IntegerFormat: Returns a formatted string representation of the provided integer. **format(int)** - Method in interface sbrt.shell.text.IntegerFormat: Returns a formatted string representation of the provided integer. **format(Integer)** - Method in class sbrt.shell.text.IntegerFormatV1: Returns a formatted string representation of the provided integer. **format(int)** - Method in class sbrt.shell.text.IntegerFormatV1: Parses the provided string and returns its corresponding integer. **format(Set<? extends E>)** - Method in interface sbrt.shell.text.LinkedHashSetFormat: Returns a formatted string representation of the provided set. **format(List<? extends E>)** - Method in interface sbrt.shell.text.ListFormat: Returns a formatted string representation of the provided list. **format(Long)** - Method in interface sbrt.shell.text.LongFormat: Returns a formatted string representation of the provided long. **format(long)** - Method in interface sbrt.shell.text.LongFormat: Returns a formatted string representation of the provided long. **format(Long)** - Method in class sbrt.shell.text.LongFormatV1: Returns a formatted string representation of the provided long. **format(long)** - Method in class sbrt.shell.text.LongFormatV1: Parses the provided string and returns its corresponding long. **format(Interval)** - Method in class sbrt.shell.text.math.BoundedIntervalFormatV1: Returns a formatted string representation of the provided interval. **format(Double)** - Method in interface sbrt.shell.text.math.CoeffFormat: Returns a formatted string representation of the provided coefficient. **format(double)** - Method in interface sbrt.shell.text.math.CoeffFormat: Returns a formatted string representation of the provided coefficient. **format(Double)** - Method in class sbrt.shell.text.math.CoeffFormatV1: Returns a formatted string representation of the provided coefficient. **format(double)** - Method in class sbrt.shell.text.math.CoeffFormatV1: Returns a formatted string representation of the provided coefficient. **format(Double)** - Method in class sbrt.shell.text.math.CoeffFormatV2: Returns a formatted string representation of the provided coefficient. **format(double)** - Method in class sbrt.shell.text.math.CoeffFormatV2: Returns a formatted string representation of the provided coefficient. **format(TauResult)** - Method in class sbrt.shell.text.math.CorrResFormatterV1: Returns a formatted string representation of the provided correlation results. **format(DirectedGraph.Edge<N>)** - Method in class sbrt.shell.text.math.DirEdgeFormatV1: Returns a formatted string representation of the provided edge. **format(S)** - Method in class sbrt.shell.text.math.IndexPairFormatV1: Returns a formatted string representation of the provided index pair. **format(Interval)** - Method in class sbrt.shell.text.math.IntervalFormatV1: Returns a formatted string representation of the provided interval. **format(LinearComb.Term<? extends V>)** - Method in class sbrt.shell.text.math.LinCombTermFormatV1: Returns a formatted string representation of the provided term. **format(LinearComb.Term<? extends V>)** - Method in class sbrt.shell.text.math.LinCombTermFormatV2: Returns a formatted string representation of the provided term. **format(LinearComb<V>)** - Method in interface sbrt.shell.text.math.LinearCombFormat: Returns a formatted string representation of the provided linear combination. **format(LinearComb<V>)** - Method in class sbrt.shell.text.math.LinearCombFormatV1: Returns a formatted string representation of the provided linear combination. **format(LinearComb<V>)** - Method in class sbrt.shell.text.math.LinearCombFormatV2: Returns a formatted string representation of the provided linear combination. **format(LinearComb.Term<? extends V>)** - Method in interface sbrt.shell.text.math.LinearCombTermFormat: Returns a formatted string representation of the provided term. **format(E)** - Method in interface sbrt.shell.text.math.MathExprFormat: Returns a formatted string representation of the provided linear combination. **format(S)** - Method in class sbrt.shell.text.math.MatrixElementFormatV1: Returns a formatted string representation of the provided matrix element. **format(S)** - Method in class sbrt.shell.text.math.SenseFormat: Returns a formatted string representation of the provided optimization sense. **format(String)** - Method in class sbrt.shell.text.NonEmptyStringFormat: Returns the provided string. **format(Double)** - Method in interface sbrt.shell.text.PercentFormat: Returns a formatted string representation of the provided percentage. **format(double)** - Method in interface sbrt.shell.text.PercentFormat: Returns a formatted string representation of the provided percentage. **format(double)** - Method in class sbrt.shell.text.PercentFormatVersion1: Returns a formatted string representation of the provided percentage. **format(Double)** - Method in class sbrt.shell.text.PercentFormatVersion1: Returns a formatted string representation of the provided percentage. **format(List<E>)** - Method in class sbrt.shell.text.SimpleListFormat: Returns a formatted string representation of the provided list. **format(Set<E>)** - Method in class sbrt.shell.text.SimpleSetFormat: Returns a formatted string representation of the provided set. **format(String)** - Method in class sbrt.shell.text.StringFormat: Returns the provided string. **format(Boolean)** - Method in class sbrt.shell.text.TrueFalseFormat: Returns "true" or "false" depending on the provided boolean value. **format(boolean)** - Method in class sbrt.shell.text.TrueFalseFormat: Returns "true" or "false" depending on the provided boolean value. **format(Boolean)** - Method in class sbrt.shell.text.YesNoFormat: Returns "Yes" or "No" depending on the provided boolean value. **format(boolean)** - Method in class sbrt.shell.text.YesNoFormat: Returns "Yes" or "No" depending on the provided boolean value. **formatElement(MatrixElement<? extends R, ? extends C, Double>)** - Method in class sbrt.shell.text.math.MatrixElementFormatV1: Returns a formatted string representation of the provided matrix element. **FormatException** - Exception in sbrt.shell.text: This exception type is used to indicate a formatting error has ocurred. **FormatException(String)** - Constructor for exception sbrt.shell.text.FormatException: Constructs a new format exception with the provided error message. **FormatException(String, Throwable)** - Constructor for exception sbrt.shell.text.FormatException: Constructs a new format exception with the provided error message and cause. **formatKey(S)** - Method in class sbrt.shell.text.AbstractMapFormat: Returns a formatted string representation of the provided key. **formatKey(S)** - Method in class sbrt.shell.text.ColonMapEntryFormatV1: Returns a formatted string representation of the provided key object. **formatKey(S)** - Method in class sbrt.shell.text.EqualsMapEntryFormatV1: Returns a formatted string representation of the provided key object. **formatKey(String)** - Method in class sbrt.shell.text.fba.FbaOptFileLineFormatV1: Returns a formatted string representation of the provided key. **formatKey(S)** - Method in interface sbrt.shell.text.MapEntryFormat: Returns a formatted string representation of the provided key. **formatKey(T)** - Method in interface sbrt.shell.text.MapFormat: Returns a formatted string representation of the provided key. **formatKeys(Set<? extends K>)** - Method in class sbrt.shell.text.AbstractMapFormat: Returns a formatted string representation of the provided set of keys. **formatKeys(Set<? extends String>)** - Method in class sbrt.shell.text.fba.FbaOptFileLineFormatV1: Returns a formatted string representation of the provided set of keys. **formatKeys(Set<? extends K>)** - Method in interface sbrt.shell.text.MapFormat: Returns a formatted string representation of the provided set of keys. **formatPair(S, T)** - Method in class sbrt.shell.text.AbstractMapEntryFormat: Returns a string representation of the provided key-value pair. **formatPair(Map.Entry<? extends K, ? extends V>)** - Method in class sbrt.shell.text.AbstractMapEntryFormat: Returns a formatted string representation of the provided map entry. **formatPair(S, T)** - Method in class sbrt.shell.text.AbstractMapFormat: Returns a string representation of the provided key-value pair. **formatPair(Map.Entry<? extends K, ? extends V>)** - Method in class sbrt.shell.text.AbstractMapFormat: Returns a formatted string representation of the provided map entry. **formatPair(S, T)** - Method in class sbrt.shell.text.fba.FbaOptFileLineFormatV1: Returns a string representation of the provided key-value pair. **formatPair(Map.Entry<? extends String, ? extends Double>)** - Method in class sbrt.shell.text.fba.FbaOptFileLineFormatV1: Returns a formatted string representation of the provided map entry. **formatPair(S, T)** - Method in interface sbrt.shell.text.MapEntryFormat: Returns a formatted string representation of the provided key-value pair. **formatPair(Map.Entry<? extends K, ? extends V>)** - Method in interface sbrt.shell.text.MapEntryFormat: Returns a formatted string representation of the provided key-value pair. **formatPair(S, T)** - Method in interface sbrt.shell.text.MapFormat: Returns a formatted string representation of the provided key-value pair. **formatPair(Map.Entry<? extends K, ? extends V>)** - Method in interface sbrt.shell.text.MapFormat: Returns a formatted string representation of the provided key-value pair. **formatPair(IndexPair<? extends R, ? extends C>)** - Method in class sbrt.shell.text.math.IndexPairFormatV1: Returns a formatted string representation of the provided index pair. **Formatter**<T> - Interface in sbrt.shell.text: This interface is used to represent *formatters*. **formatValue(T)** - Method in class sbrt.shell.text.AbstractMapFormat: Returns a formatted string representation of the provided value. **formatValue(T)** - Method in class sbrt.shell.text.ColonMapEntryFormatV1: Returns a formatted string representation of the provided value object. **formatValue(T)** - Method in class sbrt.shell.text.EqualsMapEntryFormatV1: Returns a formatted string representation of the provided value object. **formatValue(T)** - Method in class sbrt.shell.text.fba.FbaOptFileLineFormatV1: Returns a formatted string representation of the provided value. **formatValue(T)** - Method in interface sbrt.shell.text.MapEntryFormat: Returns a formatted string representation of the provided value. **formatValue(T)** - Method in interface sbrt.shell.text.MapFormat: Returns a formatted string representation of the provided value. **formatValues(List<? extends V>)** - Method in class sbrt.shell.text.AbstractMapFormat: Returns a string representation of the provided list of values. **formatValues(Map<? extends K, ? extends V>)** - Method in class sbrt.shell.text.AbstractMapFormat: Returns a string representation of the values in the provided map formatted as a list. **formatValues(List<? extends Double>)** - Method in class sbrt.shell.text.fba.FbaOptFileLineFormatV1: Returns a string representation of the provided list of values. **formatValues(Map<? extends String, ? extends Double>)** - Method in class sbrt.shell.text.fba.FbaOptFileLineFormatV1: Returns a string representation of the values in the provided map formatted as a list. **formatValues(List<? extends V>)** - Method in interface sbrt.shell.text.MapFormat: Returns a string representation of the provided list of values. **formatValues(Map<? extends K, ? extends V>)** - Method in interface sbrt.shell.text.MapFormat: Returns a string representation of the values in the provided map formatted as a list. **FORWARD\_SLASH** - Static variable in class sbrt.shell.text.SpecialChars: `/` **freeVariables()** - Method in class sbrt.kernel.math.algebra.HashLinearSystemSolution: Returns the number of free variables in this solution. **freeVariables()** - Method in interface sbrt.kernel.math.algebra.LinearSystemSolution: Returns the number of free variables in this solution.

---


|  |  |  |
| --- | --- | --- |
| **Overview** \| Package \| Class \| Use \| **Tree** \| **Deprecated** \| **Index** \| **Help** | |  |
| **PREV LETTER**   **NEXT LETTER** | **FRAMES**    **NO FRAMES**     **All Classes** |  |


A B C D E F G H I J K L M N O P Q R S T U V W X Y Z 

---
